# Market segmentation in urban tourism: A study in Latin America

**DOI:** 10.1371/journal.pone.0285138

**Published:** 2023-05-18

**Authors:** Mauricio Carvache-Franco, Otto Regalado-Pezúa, Gabriela Sirkis, Orly Carvache-Franco, Wilmer Carvache-Franco

**Affiliations:** 1 Universidad Espíritu Santo, Samborondón, Ecuador; 2 ESAN Graduate School of Business, Universidad ESAN, Surco, Lima, Peru; 3 Departamento de Ciencias Empresariales, Universidad del Cema, Buenos Aires, Argentina; 4 Facultad de Economía y Empresa, Universidad Católica de Santiago de Guayaquil, Guayaquil, Ecuador; 5 Escuela Superior Politécnica del Litoral, ESPOL, Facultad de Ciencias Sociales y Humanísticas, Guayaquil, Ecuador; University of Naples Federico II: Universita degli Studi di Napoli Federico II, ITALY

## Abstract

This study aims to analyze the different segments of urban tourism demand. The data were collected in Mexico City, Lima, Buenos Aires, and Bogota, and a K-means clustering method was used to find the segments. The results showed three segments: the first cluster grouped tourists interested in enjoying lodging and restaurant services; the second included visitors seeking multiple attractions, who were the most willing to recommend the destinations; finally, the third was composed of passive tourists, not drawn to the attractions of these cities. This study contributes to the literature by offering evidence of urban tourism segmentation in Latin American cities, which has been scarcely researched. Furthermore, it sheds light on this topic by finding a segment not previously described in the literature ("multiple attractions"). Finally, this study offers practical implications for managers of tourism companies to plan and improve the competitiveness of destinations based on the different segments found.

## 1. Introduction

Urban tourism has experienced continuous growth [1] to the point of becoming a key factor in the economy of cities. Urban destinations have adapted to their visitors’ needs [2] by making cities use their historical and physical attractions, complemented by contemporary events and the participation of other economic sectors, to entice a larger influx of tourists [3]. In addition, cities can take advantage of their appeal to foster entertainment and cultural tourism as the primary motivations of domestic and international visitors to urban destinations [4].

Urban tourism leads to economic progress, develops cultural and social aspects of people’s lives and well-being [5], and has a multiplier effect that impacts related industries and the economy of the region [6]; however, it has not received due attention in cities despite its contribution to economic development, history, and culture [7].

Market segmentation has been used to identify niche markets for tourism products and services [8]. It offers crucial advantages considering that operators worldwide experience clear pressures to ensure that consumers receive the experiences they anticipate [9]. Demand segmentation has also been studied extensively over time [8]; it helps managers identify critical visitor elements and information channels that can be used to target desired customer groups [10]. However, efficiency in targeted promotion programs is hampered by the lack of information on different segments [8].

The literature on segmentation in urban tourism is scarce. In addition, in the context of urban recreation and tourist perceptions, not much is yet known [11]. Previous studies have not delved into finding the segments according to the attractions that tourists have in urban destinations. Being this type of tourism growing, so the tourist infrastructure and the attractions of the cities should be improved according to the attractions that the different segments prefer. This is an important topic to be analyzed. For this reason, this study aims to analyze the different segments of urban tourism demand in Latin American cities using a sample from Mexico City, Lima, Buenos Aires, and Bogota. As a result, it will help tourism-related institutions better plan for the different segments of urban tourism and provide more specialized services that improve tourists’ return and benefit the destination, its sustainability, and the community.

## 2. Literature review

The dynamics of urban tourism can be understood through the tourist attractions of cities [12]. These tourist attractions in urban destinations are of different kinds. Urban infrastructure, for example, is composed of monuments and buildings that generally reflect the historical heritage of the destination and its culture, showing typical characteristics of the cities [13–17], and other cultural attractions [4]. Some cities also have important museums that entice tourists to their culture and history [16, 18].

Cities have fostered urban tourism by complementing their development with urban gardens, which charm visitors who appreciate nature and tranquility [19]. Other important tourist attractions in cities are environmentally sustainable spaces, especially for those tourists who value the environment and sustainability [20]. Also, visitors prefer public spaces they can share with others, the signage of places that facilitate travel, and the location of places to visit [21].

In addition to urban infrastructure and public spaces, important attractions in cities include entertainment and leisure activities [22] and destination-specific gastronomy [23]. Tourists are also drawn to shopping malls, stores, and commercial services [24, 25].

Tourists’ spending [26, 27], lifestyle [28], smart digital technologies within urban infrastructures [29, 30], sports in certain seasons and weather [31], and air pollution [32] are other factors in cities that can affect tourists’ intention to visit destinations.

Segmentation is the primary method to decide which groups to target, determine how to use resources more efficiently, and evaluate different competitive strategies [33]. Tourist segmentation allows tourism service providers to create preferred and valued products and services in destination markets [34]. According to Woodside & Martin [35], demand segmentation offers essential information for tourism service providers regarding the most relevant targets and markets to cover. For Ho et al. [33], market segmentation represents the decisive criterion for determining which customer groups to contact.

Segmentation studies on urban tourism have been carried out from different perspectives. For example, Fraiz et al. [36] considered the pull and push motivational factors of urban tourists and identified three segments: "health, novelty, and cultural heritage" seekers, in which cultural factors, urban leisure, and nightlife predominate; "adventure and fun" seekers, a group that wants to experience new sensations, get out of the routine and take part in urban leisure and nightlife; finally, "professionals and health seekers neutral to pull factors" is a group that wants to get out of the routine, and is more attracted to landscapes and natural wealth rather than nightlife. In addition, Chebli et al. [11] conducted urban tourism segmentation according to the personal factors of international tourists in destinations. They found two important clusters: tourists motivated by "leisure" and driven by the price of services, architecture and urban environment, public places, safety, diversity of accommodation, and hospitality; and tourists traveling for "business and professional activities", motivated by fairs, congresses, technology, accessibility to health services, stores, prices of services, diversity of transportation, accommodation, and hospitality.

With a similar perspective, Vargas et al. [28] carried out lifestyle segmentation in urban tourism and determined four segments: the first cluster, "the socials", included tourists mainly motivated by cultural activities, night visits, and shopping; the second, the "activists", was composed of visitors interested in society and the environment, cultural and recreational activities; the third cluster called "cautious" comprised tourists who value privacy and peace and show interest in society; finally, "the adolescents" included tourists interested in sports and movies, music, and technology. Pulido-Fernández et al. [37] segmented the demand according to the main activities carried out in 14 cultural-urban destinations of Andalusia, Spain. They found three important clusters: "cultural" tourists, whose primary motivation was to visit the cultural sites of the city; tourists seeking "events", motivated by leisure and entertainment in cities, such as concerts, shows, etc.; finally, the "gastronomic" tourist was driven by the gastronomy of the destinations. Similarly, Romão et al. [17] segmented tourists according to their motivations and satisfaction with urban tourism and identified three clusters. First, the group of tourists with "cultural motives" was interested in festivities and cultural events of the destination; second, tourists with "business" motives wanted to attend conferences, congresses, and business meetings; finally, visitors driven by "entertainment" in the cities generally looked for fun and good times.

Tourist segmentation can be based on the visitor’s movement patterns within the cities, considering places such as parks, gardens, green areas, etc. [38], and geographically, according to the regions visited in the destination [39]. In addition, regarding distance decay, visitors can be segmented into short-and long-haul tourists whose travel motivations, behaviors, and preferences differ [40]. Other criteria used to segment the travel market are the place of residence, accommodation type, and VRF (visiting friends and relatives) type [41].

Likewise, it is essential to point out the possible relationship between tourism segmentation, emissions, and transport. The influence of mobility and climate change on the intention of tourists to visit a destination [42] can affect tourism segmentation. In their study, Steiger et al. [43] state that the consequences of climate change on the infrastructure of tourist attractions would modify the preferences of tourists to visit the places. It divides tourists based on two characteristics: i) tourists are susceptible to changes in the infrastructure of the tourist site, and ii) tourists are indifferent to changes in the infrastructure of the tourist site. In addition, Cavallaro et al. [44] reveal that in certain areas, such as coastal cities, climate change can negatively affect the demand for tourist places over the years.

Moreover, a dual relationship between tourism, mobility, and climate change has been evidenced. Albalate and Bel [45] points out that ignoring negative externalities, such as the congestion effect that arises from the relationship between tourism and transport, cancels people’s intention to visit. It divides tourists into visitors who prefer an efficient and comfortable local transport service and tourists who do not consider these characteristics necessary. Moreover, Albalate and Fageda [46] also state the importance of the efficiency of connections between the airport and tourist destinations. Table 1 presents the different types of segmentation in urban tourism found in the literature.

**Table 1:** Types of segmentation in urban tourism.

Segmentation type	Segments	Author
Pull and push motivational	(1) Health, novelty, and cultural heritage (2) Adventure and fun (3) Professionals and health seekers neutral to pull factors	Fraiz et al. [36]
Personal factors of international tourists in destinations	(1) Tourists motivated by "leisure" (2) Business and professional activities	Chebli et al. [11]
Lifestyle segmentation	(1) The socials (2) Activists (3) Cautious (4) The adolescents	Vargas et al. [28]
Segmented according to the main activities carried out	(1) Cultural (2) Events (3) Gastronomic	Pulido-Fernández et al. [37]
According tourist motivations and satisfaction	(1) Cultural motives (2) Business (3) Entertainment	Romão et al. [17]
Visitor’s movement patterns within the cities	(1) Parks (2) Gardens (3) Green areas	Deng & Andrada [38]

Although some studies have addressed urban tourism segmentation, as mentioned above, cities have different attractions that interest diverse groups of tourists [47]. Latin American cities, for example, have particular characteristics and types of urban attractions at a historical and cultural level, urban infrastructure complemented by local gastronomy, hotels, and activities such as concerts, events, congresses, conventions, and nightlife, which call the attention of different urban tourists. Therefore, considering the characteristics of Latin American cities, there is a gap in the literature about which segments of international tourists visit these urban destinations.

This study aims to shed light on this topic and contribute to the gap in the literature on urban tourist segments by analyzing the different clusters of urban tourism demand. Hence, the following research questions are posed: RQ1: What are the segments of tourists that visit urban destinations in the cities of Mexico City, Lima, Buenos Aires, and Bogota? and RQ2: What is the relationship between these segments of tourists and the recommendation to travel to these destinations?

## 3. Methodology

This research administered a questionnaire used in previous studies [47–49]. The study population was defined as men and women older than 18 years who had traveled to Mexico City, Buenos Aires, Lima, and Bogotá between January 2018 and March 2020. These cities were chosen because they are the ones with the largest population in Hispanic America [50]. We have taken this factor as an indicator to determine its size and importance. In turn, it is relevant to investigate cities with similar dimensions as a whole, so the research was limited to these four cities and not to all Latin American cities due to the diversity that it would imply.

The survey was conducted between September and December 2020, in an online and self-administered format. Convenience sampling was used to select the 599 participants. The number of positive responses was 32%. The number of cases was sufficient since it is suggested that for a population with a variance of 50% [51], taking a confidence level of 95% and a margin of error of 5%, the number should be 384 or higher [52]. The questions were rotated, as well as the appearance of the cities visited in the period of time investigated. In this way, biases due to the order and influence of the previous questions were avoided.

Written informed consent was requested in the questionnaire. The project was ethically approved by the ESAN University of Peru. Data collection was conducted in 2020 using a questionnaire with 16 questions. The first section consisted of questions that analyzed the sociodemographic and behavioral profile of tourists. The second section consisted of 14 items that evaluated the attractions in urban tourism. This section consisted of Likert-type questions, where 1 was the least important and 5 was the most important. The third section consisted of questions that analyzed the willingness to recommend urban destinations. This section was made up of a Likert-type scale, where 1 was unwilling and 10 was very willing. The sample included 599 surveys, and the SPSS software version 22 was used to process the data. In addition, Cronbach’s alpha was used to estimate the internal consistency of the attractions scale, made up of 14 items. A value greater than 0.80 indicates that the scale has a high internal consistency [53–55]. In this study the value of 0.924 was obtained.

The K-means clustering method was used to find the segments. Kruskal Wallis H test [56] was applied to analyze the significant differences between the means of the three groups. In addition, the Mann-Whitney U test [57] was performed to determine the significant difference between the two groups. Finally, the Net Promoter Score (NPS) indicator was used to find the relationship between the segments and their intentions to recommend the travel destination on a scale from 1 to 10, with scores from 9 to 10 for promoters, 7 to 8 for passive (or neutral), and 1 to 6 for detractors.

## 4. Study area

The following is a description of the four capital cities with the largest Spanish-speaking population in Latin America, where the fieldwork was conducted:

### 4.1. Mexico city

The capital of the United States of Mexico has a population of 22.09 million inhabitants [58]. In 1987, due to its concentration of a diversity of colonial buildings, including temples, museums, galleries, theaters, and cultural centers, its historic center was declared a World Heritage Site by UNESCO [59, 60]. In addition to the monuments and urban architecture, its cultural richness can be seen in the city’s daily life, art, festivities, and varied gastronomy. Furthermore, this modern metropolis in the northern hemisphere with a large domestic market is a privileged venue for various congresses and events. The Ministry of Tourism [61] indicated that last year the Mexican capital received 6.8 million tourists, leaving an economic revenue of USD 3.15 million.

### 4.2. Lima city

Lima, the capital of the Republic of Peru, has a population of 11.05 million inhabitants [58]. Visitors walking the streets of this city can discover the legacy of pre-Hispanic cultures, colonial monuments, architecture, and its history and tradition [62]. For example, the Plaza Mayor, located in the historic center, is surrounded by buildings with French neo-baroque influence, such as the Government Palace, the Cathedral, the Archbishop’s Palace, and the Church of the Sagrario. Furthermore, the country’s economic growth has allowed the development of hotels and convention centers, which gave rise to the impulse of MICE tourism. Another attraction to consider is Peruvian gastronomy, which is highly demanded and recognized, triggering the opening of new restaurants and the consequent supply chain development. In 2020, Lima received 507,883 foreign tourists, far from the pre-pandemic figures of over 2.6 million visitors [63].

### 4.3. Buenos aires city

According to Statista [58], the federal capital of Argentina, including Greater Buenos Aires, has a population of 15.37 million inhabitants. Buenos Aires possesses a set of attractions and architectural designs that qualify it as a modern and cosmopolitan city. Its urban profile is markedly eclectic, mixing art deco, art nouveau, neo-Gothic, and French Bourbon styles attributed to immigration [64]. Its public spaces and green areas make up its urban environment and are part of its tourist attractions and appeal [65]. Similarly, the customs of its population, the tango and its sensuality, the gaucho and its tradition, its places, and the literature with its characters make this capital the gateway to the country that in 2019 received 2.9 million international tourists and left a revenue of USD 1.83 million [66].

### 4.4. Bogota city

The Colombian capital has a population of 11.34 million inhabitants [58]. According to ProColombia [67], Bogota is one of the country’s most important cultural and recreational centers. In addition to concentrating most of the cultural tourist attractions, such as the Gold Museum or the Colon Theater, located in the La Candelaria neighborhood, the historic center is mixed with the modernity of shopping malls, universities, and residential complexes. Visitors appreciate the diversity of places to visit, the museums, and traditional neighborhoods, with narrow streets and colonial mansions; they also enjoy the *ajiaco*, a typical dish of the local cuisine. As a result, this city attracts significant inbound tourism and also has a good outbound tourism flow. In fact, in 2021, Bogota received almost four hundred thousand of the approximately one million tourists that arrived in the country [68].

## 5. Results

Table 2 shows the descriptive results obtained. The predominant groups of international tourists were those between 18 and 45 years old and those between 46 and 60 years old. In addition, 94.65% of the tourists came from other countries in the Americas, excluding the USA and Canada, which represented 2.33%, while Europe accounted for 2.67%.

**Table 2:** Sociodemographic characteristics.

Demographics	Categories	N = 599	%
Age	18–45 years	285	47.57
	46–60 years	276	46.07
	Greater than 60	38	6.34
Origin	USA and Canada	14	2.33
	Other American countries	567	94.65
	Europe	16	2.67
	Asía	2	0.33

### 5.1. Urban tourism segmentation

The K-means clustering method was used to perform the segmentation of urban tourism. To find the number of segments, the dendrogram of the hierarchical analysis was used, which resulted in three groups. The options of 2 and 4 conglomerate were analyzed and it was found that they were not the ones indicated. In the option of 4 clusters, two of them were found to be similar, that is, they did not present significant differences. Likewise, with the 2-cluster option, similarities were also found between the two segments. Therefore, the option of three conglomerates was finally decided. The Kruskal Wallis H test indicated a significant difference between the means of the three segments. The Mann-Whitney U test also showed a significant difference for each of the groups. Table 3 presents the results,

**Table 3:** Urban tourism segmentation.

Variable	Lodging and restaurant services	Multiple attractions	Passive tourism	Kruskal Wallis H	Sig.	Mann-Whitney U
Urban atmosphere	3.7	4.4	2.1	209.481	0.000	All (1–2; 1–3:2–3)
Urban architecture	3.4	4.4	1.9	257.710	0.000	All (1–2; 1–3:2–3)
Monuments and historical sites	3.6	4.6	2.1	219.795	0.000	All (1–2; 1–3:2–3)
Public spaces, parks, and gardens	3.5	4.5	2.1	235.830	0.000	All (1–2; 1–3:2–3)
Accommodations and restaurants	4.3	4.8	3.0	146.258	0.000	All (1–2; 1–3:2–3)
Public services	3.2	4.1	1.7	198.497	0.000	All (1–2; 1–3:2–3)
Tourist information	3.4	4.5	1.8	247.323	0.000	All (1–2; 1–3:2–3)
Shops and commercial services	3.4	4.2	2.1	172.225	0.000	All (1–2; 1–3:2–3)
Museums and art galleries	3.3	4.4	1.7	236.268	0.000	All (1–2; 1–3:2–3)
Access and signage	3.2	4.3	1.7	230.729	0.000	All (1–2; 1–3:2–3)
Excursions	3.0	4.3	1.7	253.118	0.000	All (1–2; 1–3:2–3)
Festivals and events	2.9	4.1	1.7	241.449	0.000	All (1–2; 1–3:2–3)
Theaters, concerts, and nightlife	3.2	4.3	1.7	222.129	0.000	All (1–2; 1–3:2–3)
Fairs, conventions, and exhibitions	3.1	4.2	2.0	179.976	0.000	All (1–2; 1–3:2–3)

According to the results in Table 2, the first segment was called "lodging and restaurant services" and was made up of tourists attracted by the hotel infrastructure and restaurants; this cluster comprised 38.6% of the sample. The second segment was called "multiple attractions" and gathered tourists drawn by all the attractions analyzed in this study; this group included 50.6% of the respondents. Finally, the third cluster, named "passive tourism," showed tourists with a low level of attraction for all the variables; this segment accounted for 10.8% of the sample.

### 5.2. Segmentation and intentions to recommend the travel destination

The Net Promoter Score (NPS) indicator was used to measure the willingness of tourists to recommend the destination. The crossover was made between the three segments found and the question that analyzes the willingness of tourists to recommend the destination, made up of a 10-point Likert scale. Pearson’s Chi square test was used to find the significant relationship between the variables. The percentage of tourists who answered with 9 points was added to the percentage of tourists who answered with 10 points. This group was called promoters. Tourists who responded between 7 and 8 are named neutrals, and tourists who responded between 1 and 6 are called detractors. Table 4 shows the results.

**Table 4:** Segmentation and destination recommendation.

Variable	Lodging and restaurant services (%)	Multiple attractions (%)	Passive tourism (%)	Total (%)	χ 2	Sig.
Promoters (9,10 points)	47.5	75.7	36.4	60.6	95.938	0.000
Neutral (7, 8 points)	39.8	19.7	39.4	29.6		
Detractors (1–6 points)	12.7	4.5	24.2	9.8		
Total (%)	100	100	100	100		

According to the results in Table 4, the crossing of the three segments and the variable willingness of tourists to recommend the destination was significant (p<0.05). The "multiple attractions" segment had the highest percentage of promoters (75.7%). Therefore, this was the segment most willing to recommend the destination. The cluster "lodging and restaurant services" had a similar percentage of promoters (47.5%) and neutrals (39.8%), so this segment groups tourists willing to recommend the destination (promoters); however, they may not be entirely satisfied, would not return to the cities or would not recommend their family and friends to visit the place. On the other hand, the "passive tourists" cluster had a higher percentage of neutrals (39.4%) than promoters (36.4%), something to be expected due to their low attachment to the destination.

Another view is that of the detractors. It can be seen that the segment "passive tourism" concentrated 24.2% of the detractors, the cluster "lodging and restaurant services" 12.7%, and the group "multiple attractions" 4.5%. The "multiple attractions" segment had the lowest number of detractors; however, since it was the most satisfied segment, it would be interesting to address, in future studies, the reasons behind that result. In addition, these findings point to the need for improvements in all tourist attractions to motivate the promoters of the "multiple attractions" cluster, who are the most willing to recommend these destinations. Likewise, positive changes should be incorporated in lodging and restaurants to attract the promoters of the "lodging and restaurant services" segment, who are also eager to recommend these destinations.

### 5.3. Segmentation and the purpose of travel

A cross-checking of variables between segments and the purpose of travel was performed. The results are presented in Table 5.

**Table 5:** Segmentation and the purpose of travel.

Variable	Lodging and restaurant services (%)	Multiple attractions (%)	Passive tourism (%)	Total (%)
Business, Conventions, Professional Activities	51.3	44.7	65.2	49.4
Leisure Tourism	28.8	35.3	19.7	31.1
Visiting family or friends	13.6	12.3	13.6	12.9
Studies	1.3	3.2		2.1
Others	5.1	4.5	1.5	4.4
Total (%)	100.0	100.0	100.0	100.0

According to Table 5, the "passive tourism" segment, with 65.2%, was the cluster with the highest percentage of tourists traveling for business, conventions, and professional activities. This group was followed by the "lodging and restaurant services" segment, with 51.3%. Finally, the "multiple attractions" cluster had the highest percentage of tourists traveling for leisure tourism (35.3%). Therefore, improvements should be made in the attractions related to business, conventions, professional activities, and leisure tourism.

It is also relevant to note that the "multiple attractions" segment, which is the most willing to recommend, obtained a high percentage (35.3%) in leisure tourism. However, the business purpose as a reason for the trip continues to predominate.

### 5.4. Segmentation and first-time visitors to the destination

A crossover of variables between segments and first-time visitors was performed. The results are shown in Table 6.

**Table 6:** Segmentation and first-time visitors.

Variable		Lodging and restaurant services (%)	Multiple attractions (%)	Passive tourism (%)	Total (%)
First-time visitors to the destination	No	71.2	69.3	66.7	69.7
	Si	28.8	30.7	33.3	30.3
Total (%)		100.0	100.0	100.0	100.0

According to the results in Table 5, the "lodging and restaurant services" segment, with 71.2%, was the group with the highest percentage of tourists who have already visited the destinations more than once, so they have already returned to these places. This cluster was followed by the "multiple attractions" group with 69.3%. While the "passive tourism" segment, with 66.7%, was the one with the least revisits and the highest percentage of first-time visitors. This means that the attractions related to lodging and restaurants should be improved, followed by the other attractions, to increase tourists’ return intentions. Table 7 shows a characterization of the three segments.

**Table 7:** Characterization of the segments.

Variables	Lodging and restaurant services	Multiple attractions	Passive tourism
Level of attractions in the destination	They are attracted to accommodations and restaurants	They are attracted by all the attractions of the destination	Low level of attraction in the destination
By their level of willingness to recommend the destination	They are promoters and neutrals. They recommend the destination. But, they may be prone to switch to the competition.	Mostly promoters. They are the ones who recommend the destination the most and the most loyal. They are not likely to move to other destinations that are part of the competition.	Promoters, neutrals and detractors. They are the ones with the highest percentage of detractors. They are the ones who least recommend the destination. With high probabilities not to return to the destination
For your travel purpose	They travel for business, but a smaller percentage travel for leisure	They travel for both business and leisure	They travel only for business
Tourists on their first visit or tourists who have already returned to the destination	Higher percentage of tourists who have returned to the destination in relation to other groups	Higher percentage of tourists who have returned to the destination. Lower percentage of tourists on first visit	Higher percentage of tourists on first visit in relation to other groups

## 6. Discussion

This research aimed to analyze the different segments of urban tourism demand in Mexico City, Lima, Buenos Aires, and Bogota. Concerning question RQ1, the results showed three segments: the first cluster gathered tourists attracted by the lodging and restaurant services, the second group comprised tourists drawn by all attractions in these destinations, and the third segment included tourists with a low level of interest toward the attractions of these cities.

The first segment of tourists was attracted by the lodging and restaurant services; these visitors generally appreciate the good hotel infrastructure in the cities and are drawn to restaurants with good ambiance, comfortable and elegant. This result is in line with those found by André Romero et al. [69] and Stepchenkova [70], who identified a segment of urban tourism motivated by leisure and entertainment. In addition, this result agrees with those found by Chebli et al. [11], Pulido-Fernández et al. [37], and Fraiz et al. [36], who described tourists attracted to leisure and entertainment in cities as an important segment.

The second segment was made up of tourists interested in multiple attractions simultaneously. In addition, this cluster was the most willing to recommend the destination, making it the most salient group. It is important to mention that no similar segment has been found in the academic literature. Other different clusters identified are the ones described by Pulido-Fernández et al. [37], called "cultural" tourists and tourists seeking "events", and by Romão et al. [17], named cultural and business-motivated tourists.

The third segment grouped passive tourists, with low levels of interest toward almost all the attractions. Similar clusters have been found in the literature by Fraiz et al. [36], who identified a group of neutral tourists not attracted by the pull factors in the cities, and by Vargas et al. [28], who established a segment of "cautious" tourists that seek peace and tranquility in the cities.

This study contributes to the academic literature by finding a segment in urban tourism called "multiple attractions", which had not previously been found in the scientific literature. The tourists that make up this segment are drawn by everything the destination has to offer, such as lodging and restaurants, urban environment, public services, stores, and commercial services, monuments, and historical sites, urban architecture, museums and art galleries, festivals and events, fairs, conventions, museums, theaters, concerts, and nightlife.

Concerning question RQ2, the segment "multiple attractions" had the highest percentage of promoters, tourists more willing to recommend the destinations. Therefore, more interest should be given to improving this segment, which probably has the most loyal tourists, more willing to revisit and recommend the destinations. These visitors enjoy tourist and cultural activities, so attention should be paid not only to tourism services in general but to cultural activities and the organization of events. It could be assumed that if the number of tourists who recommend the destination increases, the number of new tourists and their revisit intention will also increase.

The theoretical implications of this study are two-fold. First, it contributes to the literature by finding the segments of tourists that visit Mexico, Lima, Buenos Aires, and Bogota since these cities have a lot of attractions that stand out not only for their quantity but their quality, differing from other destinations. Second, this research shows the relationship between the segments of tourists and their recommendation to travel to these cities as tourist destinations.

The practical implications of this study are helpful for managers and administrators of tourism companies in Latin American cities, who can use the knowledge of the international tourist segments to design plans and improve the competitiveness of the destinations based on the characteristics of the different clusters. Furthermore, this knowledge is also useful for urban, sustainability, and city development studies that consider tourism an essential source of urban development.

The segmentation carried out in this study will help urban destinations to know the main attractions that the different segments prefer and to adapt improvements in the destinations according to these preferences.

## 7. Conclusions

Demand segmentation is a tool that serves to divide demand into various groups of tourists, improving the interest of each segment through an adequate and specific offer that increases tourist satisfaction and destination recommendation.

This study found three segments of national and international tourists in Mexico City, Lima, Buenos Aires, and Bogota. In the first segment, tourists are attracted mainly by leisure and fun. They are primarily drawn to hotel facilities and comfortable and elegant restaurants with a good atmosphere, and local and international cuisine. Likewise, this cluster showed a high percentage of tourists driven by business, conventions, and professional activities. In the second segment, tourists are motivated by all attractions in these destinations, such as urban environment, public services, stores and commercial services, lodging and restaurants, monuments, and historical sites, urban architecture, museums and art galleries, festivals and events, fairs, conventions, museums, theaters, concerts, and nightlife. In addition, this cluster comprised the highest percentage of promoters (tourists willing to recommend the destination), so it can be considered an important segment. This group was also interested in business tourism, conventions, professional activities, and leisure tourism. Finally, in the third segment, we found passive tourists with low levels of interest toward these cities’ urban attractions. This group included tourists motivated by businesses, conventions, and professional activities.

The theoretical implications of this research are its contribution to a core area. This study identified three distinct segments of tourists: the group attracted by the lodging and restaurant services, the group drawn by the multiple attractions, and the group composed of passive tourists. This information contributes to closing the gap in the literature on the segments of tourists visiting urban destinations in Latin American cities, whose urban tourist attractions have specific characteristics.

The practical implications of this research are founded on the fact that knowledge of the tourist segments traveling to these Latin American destinations can be used by managers of tourism companies and city planners, who can devise plans to improve the competitiveness of the cities based on the characteristics of the different segments. Likewise, this information can be used in urban and city development studies that consider tourism an essential economic activity or an important source of urban development.

Knowledge of the tourist segments in these Latin American cities can be used to better the supply and related processes in hotels and restaurants and enhance the customer experience, infrastructure, and complimentary services. In addition, these cities’ accommodations and gastronomic image could be improved to motivate tourists to travel. Events related to gastronomy could be organized to better position these cities and foster their international recognition. Likewise, these destinations’ architectural and cultural image should be improved through cultural events and artistic presentations that increase their appreciation at a cultural level. The organization of events and congresses should also be encouraged to showcase these cities as suitable destinations for hosting events and developing business tourism, conventions, and professional activities.

The segmentation method used in this study presents a generalization potential for future segmentation studies in urban destinations. Among the limitations of the study was the temporality with which the sample was collected. As future lines of research we propose the perception of the image of urban destinations.

## Supporting information

S1 File (SAV)
